# A small molecule inhibitor of the perinucleolar compartment, ML246, attenuates growth and spread of ovarian cancer

**DOI:** 10.1186/s40661-018-0064-2

**Published:** 2018-10-02

**Authors:** Margaux J. Kanis, Wenan Qiang, Mario Pineda, Kruti P. Maniar, J. Julie Kim

**Affiliations:** 1 Division of Gynecology Oncology, Northwestern University Feinberg School of Medicine, Chicago, IL USA; 2 Division of Reproductive Science in Medicine, Department of Obstetrics and Gynecology, Northwestern University Feinberg School of Medicine, Chicago, IL USA; 3 Department of Pathology, Northwestern University Feinberg School of Medicine, Chicago, IL USA; 4 Robert H. Lurie Comprehensive Cancer Center, Northwestern University, 303 E. Superior Street, 4-117, Chicago, IL 60611 USA

**Keywords:** Perinucleolar compartment, Ovarian cancer, ML246, Metarrestin

## Abstract

**Background:**

Ovarian cancer remains a major health problem for women as it is often diagnosed at a late stage with metastatic disease. There are limited therapeutic agents and survival rates remain poor. The perinucleolar compartment (PNC) has been shown to be associated with malignancy and is considered a surrogate phenotypic marker for metastatic cancer cells. A small molecule, ML246, was derived from a screen against PNCs. In this study, the effect of ML246 on ovarian cancer growth and spread was investigated.

**Methods:**

SKOV3 or OVCAR3 cells were treated with ML246 in vitro and PNC was visualized with immunofluorescent staining. Cell invasion was assessed using Matrigel-coated transwell systems. SKOV3 cells were xenografted orthotopically under the ovarian bursa of immunocompromised mice. Additionally, a patient derived ovarian cancer cell line was grafted subcutaneously. Mice were treated with ML246 and tumor growth and spread was assessed.

**Results:**

PNCs were prevalent in the ovarian cancer cell lines OVCAR3 and SKOV3 with higher prevalence in OVCAR3 cells. Treatment with ML246 significantly reduced PNC prevalence in OVCAR3 and SKOV3 cells. Moreover, the invasive activity of both cell lines was significantly inhibited in vitro. Orthotopic implantation of SKOV3 cells resulted in growth of the tumor on the ovary as well as spread of tumor tissues outside of the primary site on organs into the abdominal cavity. Treatment with ML246 decreased the incidence of tumors outside of the ovary. In addition, a patient-derived xenograft (PDX) line was grafted subcutaneously to monitor tumor growth. ML246 significantly attenuated growth of tumors over a 5-week treatment period.

**Conclusions:**

PNC’s are present in ovarian cancer cells and treatment with ML246 decreases invasion in vitro and tumor growth and spread in vivo. Additional studies are warranted to determine the efficacy of ML246 as an inhibitor of metastatic disease in ovarian cancer and to determine its precise mechanism of action.

## Background

Ovarian cancer is the most lethal of the gynecologic cancers with approximately 14,000 women dying annually in the United States from this disease [1]. Although most of these cancers are responsive to initial chemotherapy, relapse from remission frequently occurs, resulting in death from widespread metastatic disease. Effective therapeutics are currently lacking to successfully overcome this disease leading to the low long-term survival rates for ovarian cancer patients. Novel treatments that inhibit ovarian cancer progression are in urgent need to improve the outcomes of these patients.

Targeted therapy is changing the therapeutic landscape in oncology and agents including angiogenesis inhibitors and PARP inhibitors, alone or in combination with chemotherapies, have been shown to be promising advancements for the treatment of ovarian cancer [2]. While these agents target physiological processes such as angiogenesis and DNA repair, targeting cellular structures that form specifically in malignant cells is another approach to tackle cancer. The perinucleolar compartment (PNC) is a nuclear body, containing RNA polymerase III transcripts and polypyrimidine tract-binding protein (PTB), that is adherent to but distinct from the nucleolus [3–5]. They are observed in various metastatic solid tumors and transformed cell lines including breast, prostate, pancreatic and colorectal cancers, but absent in normal cells including embryonic stem cells [6–9]. PNC prevalence in tumors of breast and colorectal cancer were shown to be positively associated with disease severity and inversely correlated with patient survival [6, 8]. Its prevalence was high, particularly in metastatic tumors and metastatically transformed cancer cell lines, making it a potential pan-marker for metastatic progression. Although the precise function of PNCs remains unclear, studies have implicated possible roles in RNA metabolism [10–13], and thus, inhibition of PNCs could render the cancer cells unable to grow and spread by dysregulating the ribosome machinery. A compound to decrease PNC prevalence in cancer cells, ML246 (*Metarrestin*) was identified through a high throughput screen [14, 15]. Given the growth and metastatic characteristics of ovarian cancer and the urgent need to better treat this deadly disease, we investigated the effects of the novel drug, ML246 on PNC prevalence and invasive activity of ovarian cancer cells in vitro and tested its efficacy on tumor growth and spread in xenograft models.

## Methods

### Cell lines and PDX

The human ovarian cancer cell lines SKOV3 and OVCAR3 were obtained from the laboratory of Dr. Jian-Jun Wei (Northwestern University) [16]. SKOV3 was cultured in McCoy’s 5A supplemented with 10% fetal bovine serum (FBS) and 1% penicillin/streptomycin and cultured in a humidified incubator (5% CO_2_) at 37 °C. OVCAR3 cells were cultured in Dulbecco’s modified Eagle’s medium (DMEM) supplemented with FBS 20%. All cell lines were authenticated.

The PDX line used in this study was originally obtained from primary ovarian high grade serous carcinoma at the time of surgery at the Prentice Women’s Hospital of Northwestern University, Chicago, USA. This line was propagated in NSG mice and characterized as previously described [17]. For this study, frozen PDX tumor fragments at passage 3 were repropagated in NSG mice.

### Immunofluorescence for detection of PNC

SKOV3 and OVCAR3 cells were cultured on glass cover slips for 24 h. Cells were treated for 24 h with DMSO, 0.05 μM, 1 μM or 2 μM of ML246 and then fixed with 4% paraformaldehyde in PBS for 10 min, washed, and solubilized in 0.5% Triton X-100 in PBS for 5 min. Cells were incubated with anti-PTB primary antibody SH54 [3] or anti-fibrillarin (Sigma Chemical Co., St. Louis, MO), a marker for the nucleolus, for 1 h at room temperature. Cells were rinsed in PBS and then incubated with Texas red–conjugated goat anti–human or FITC-conjugated goat anti–mouse antibody for 1 h at room temperature. The coverslips were mounted onto glass slides with mounting medium containing DAPI to visualize the nucleus. Cells were examined with a fluorescent microscope (FXA; Nikon Inc., Melville, NY). Images were captured by a SenSys cooled CCD camera (Photometrics, Tucson, AZ) using Oncor Image software. PNCs were counted based on an approximate 2-fold or more intense signaling of the PTB labeling than the nucleoplasm. Cells with PNC, as well as cells with multiple PNCs, were counted in four different fields of view. This experiment was performed in triplicate.

### Cell viability and invasion assays

SKOV3 and OVCAR3 cells were treated with vehicle or ML246 for 72 h at the indicated concentrations. Cell viability was measured using the WST-1 viability assay as per the manufacturer’s instructions (Roche, Switzerland).

In vitro invasion assays were performed on SKOV3 and OVCAR3 using 24-well transwell units with polycarbonate filters (pore size: 8 μm) coated on the upper side with reconstituted basement membrane matrix, Matrigel (BD Biosciences, USA). 5 × 10^5^ cells were added to the transwell in serum free media. Media with FBS was added to the outer compartment as the chemoattractant. Cells were treated with DMSO or ML246, and cultured for 72 h at 37 °C. Cells remaining on the upper side of the transwell were scraped off with a cotton swab. Cells remaining on the underside of the membrane were fixed and stained using the commercially available, Shandon Kwik-Diff stain kit (ThermoFisher Scientific). Cells were counted in four fields and the mean + SEM was calculated. This experiment was performed in triplicate in three independent experiments.

### SKOV3 Xenografts

#### Preparation of cell pellets

SKOV3 cells were prepared in collagen pellets for intrabursal xenoplantation. Cells were suspended in a rat-tail collagen (type I) solution (BD Bioscience, San Jose, CA) at 10^6^ cells per 10 μl as previously described [16]. The cell–collagen mixture was then dropped onto a 6-well plate and incubated at 37 °C in a humidified atmosphere of 95% air and 5% CO_2_ for 30 min. The pellets were incubated overnight as floating cultures.

#### Surgical procedure for implantation of xenografts in intraovarian bursa

The implantation of xenografts into the intraovarian bursa has been previously described [16]. Briefly, nude mice (Jackson Laboratories) at 8 weeks old were used for xenografting. All animal experiments were approved by the Institutional Animal Care and Use Committee of Northwestern University. Mice were anesthetized with ketamine/xylazine (90/8 mg/kg) by intraperitoneal (IP) injection. An incision was made in the skin just laterally to the midline of the lower back, and the left ovary was exteriorized. A tiny incision was made in the bursa of the ovary with the aid of a dissecting microscope and the cell pellet was grafted into the ovarian bursa. The pellet was fixed in place due to the tension of bursa. The ovary was placed back into the body cavity, and no bleeding was noted. The incision was surgically closed.

#### Preparation of ML246 solution

ML246 solution was prepared by dissolving ML246 into 5% (*v*/v) N-methylpyrrolidine (NMP, Sigma), then adding 20% (v/v) Polyethylene glycol 400 (PEG-400, Sigma), followed by 75% (v/v) of 10% (2-Hydroxypropyl)-β-cyclodextrin (HP-β-CD, Sigma) dropwise into the tube. 200 μl of 1.5 mg/ml (for PDX) or 2.5 mg/ml (for SKOV3) of ML246 or vehicle (5% NMP, 20% PEG-400, and 75% of 10% HP-β-CD) were intraperitoneally injected into mice per 20 g body weight for the treatment dosage of 15 mg/kg or 25 mg/kg. Mice were treated once a day on the weekdays (Monday through Friday).

#### ML246 treatment in mice

Treatment commenced 3 weeks after grafting of SKOV3 cell pellets. Twelve mice received ML246 by intraperitoneal injection at 25 mg/kg on a weekday schedule for a total of 7 weeks. Ten mice received cisplatin by intraperitoneal injection as a positive control and 12 mice received vehicle as the negative control. Mice were harvested 10 weeks post-xenografting. Organs and tumor deposits were weighed, processed and preserved. H&E analysis was performed by a pathologist to confirm the presence of tumor in the ovary and in suspected metastatic lesions. Mice were monitored for behavior and activity on a daily basis, and weights obtained three times per week.

### Patient-derived xenografts

#### Subcutaneous implantation of ovarian cancer PDX line

The OVCA4P PDX tumor line from the third passage (P3) [17] was used for subcutaneous xenografting into ten-week-old female adult non-obese diabetic (NOD)-scid IL2Rγ null (NSG) mice (The Jackson Laboratory). PDX tumor tissues were cut into small fragments, approximately 2 × 2 × 2 mm. Two pieces were subcutaneously xenografted into two dorsal flanks of NSG mice, under anesthesia by intraperitoneal injection of ketamine/xylazine (90/8 mg/kg). The surgical site was shaved and disinfected with providone iodine prep pads and alcohol swab (70% isopropyl alcohol). A 1 cm incision was made in the skin at the midline of the mouse dorsum, and two separate tumor fragments were placed into the lower left and lower right of dorsal side. The skin was sutured, and mice were allowed to recover. Tumors were allowed to grow for 3 weeks. Five mice were treated with 15 mg/kg ML246 and 5 mice were treated with vehicle by intraperitoneal injection once a day on a 5-day schedule (Monday through Friday) for 5 weeks. Subcutaneous tumor size was monitored by palpation and measured with digital calipers weekly.

### Tissue processing

Tumors were fixed in modified Davidson’s solution. All fixed tumor tissues were processed, embedded in paraffin, sectioned, and then stained with H&E. Tumor histology, differentiation, invasion and metastasis were examined by a gynecologic pathologist. Extra sections and slides were reviewed for areas in which extra-ovarian tumor deposits were suspected.

### Statistical analysis

Prism/GraphPad Software (LaJolla, California) was used for all statistical analysis. PNC prevalence and invasion assays were analyzed by a one-way ANOVA followed by Tukey’s multiple comparisons test. The mean total tumor volumes of the subcutaneous xenografts were analyzed with the Mann-Whitney U test comparing vehicle and ML246 treated mice at each time point. *P* < 0.05 was considered significant.

## Results

### Effect of ML246 on PNCs in ovarian cancer cell lines

**Fig. 1:**
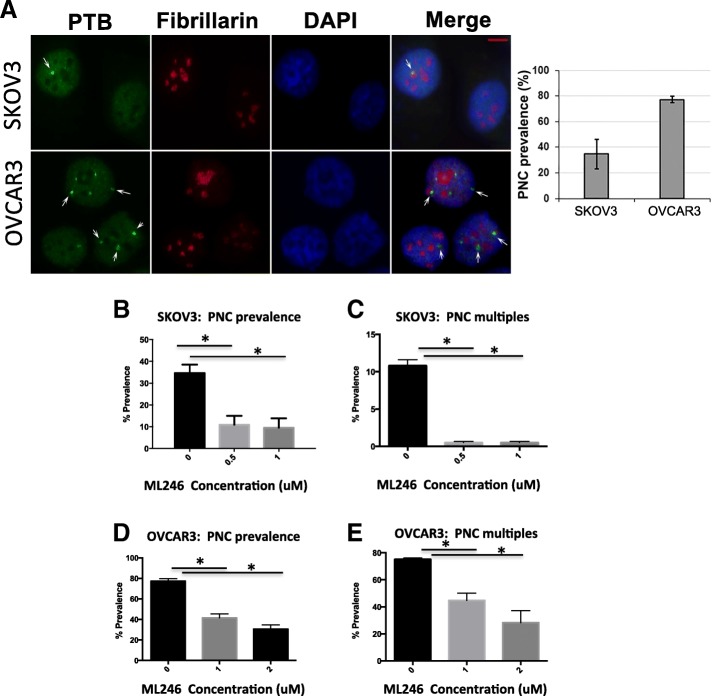
PNC prevalence in ovarian cancer cell lines. **a** Immunofluorescent staining was done in SKOV3 and OVCAR3 for PTB, a component of PNC (green, marked by arrows) that are located adjacent to nucleoli (fibrillarin staining in red) within the nucleus (DAPI staining in blue). Bar = 5 μm. **b** and **c** SKOV3 and **d** and **e** OVCAR3 cells were treated with ML246 at 0.05uM, 1uM or 2 uM or DMSO for 24 h and % PNC prevalence as well as cells carrying multiple PNCs was calculated. * *p* < 0.05

PNCs were detected in the ovarian cancer cell lines, SKOV3 and OVCAR3, using immunolabeling with SH54 antibody that specifically recognizes PTB, a key marker protein of PNCs. PNCs were present in both cell lines as seen by the bright green signal around the nucleolus, with a higher PNC prevalence in OVCAR3 cells than in SKOV3 cells (Fig. 1a). As ML246 was identified in a drug screen as a compound that effectively targeted PNCs in tumor cells [14, 18], the prevalence of PNCs was examined in SKOV3 and OVCAR3 cell lines before and after treatment with ML246. The PNC prevalence in both SKOV3 (Fig. 1b) and OVCAR3 (Fig. 1d) cells significantly decreased with ML246 treatment. The number of cells with multiple PNCs per nucleus also decreased in both SKOV3 and OVCAR3 cell lines with ML246 treatment (Fig. 1c, e). The increased incidence of multiple PNCs in the OVCAR3 cell line may reflect the aggressive nature of a serous ovarian cancer cell line, as opposed to SKOV3 which originated from an endometrioid ovarian tumor [19].

### Effect of ML246 on invasion of ovarian cancer cells in vitro

**Fig. 2:**
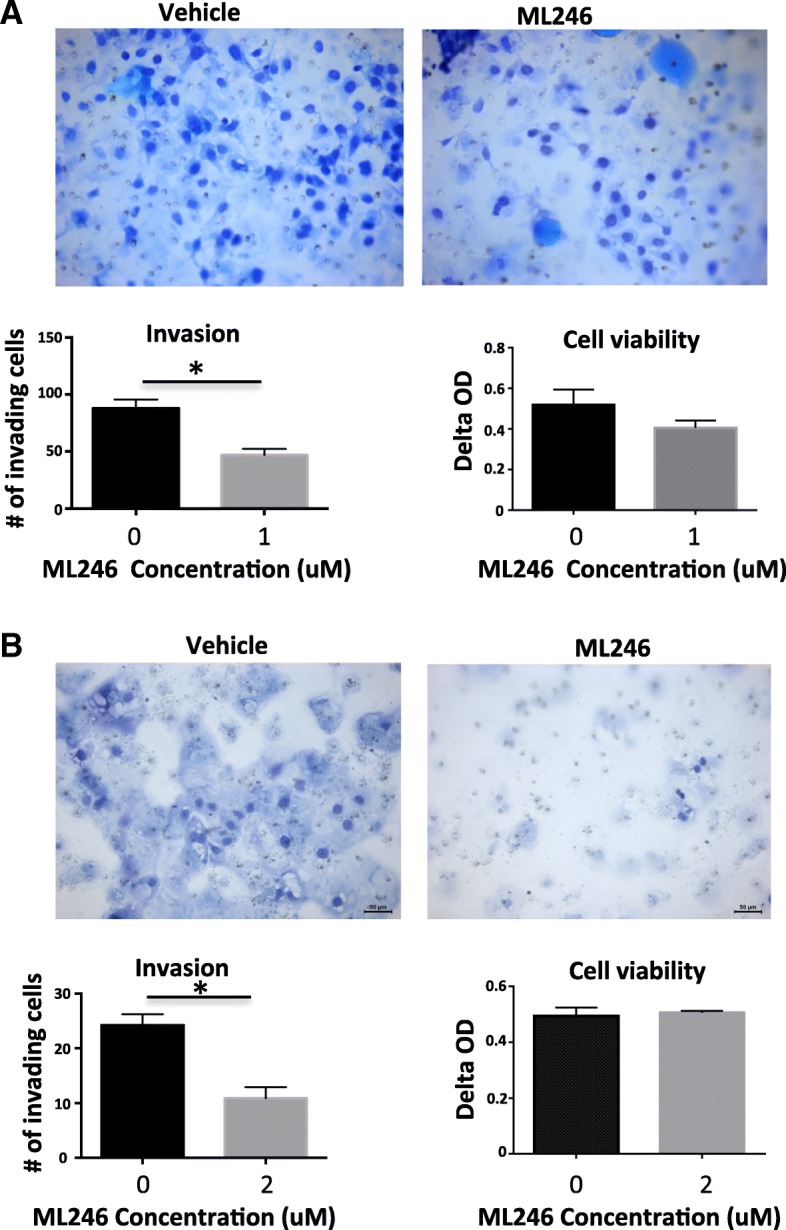
Effect of ML246 on invasive activity of ovarian cancer cells. **a** SKOV3 and **b** OVCAR3 cells were plated onto Matrigel coated invasion chambers and subsequently treated with 1uM or 2uM of ML246, respectively for 72 h. Cells that invaded and migrated through the Matrigel and porous membrane were stained and imaged at 40×. Cells were counted in four fields and the mean + SEM was calculated. This experiment was performed in triplicate in three independent experiments. WST-1 viability assay was done on cells treated under the same conditions to assess viability after ML246 treatment. *p* < 0.05

The effect of ML246 on the invasive properties of SKOV3 and OVCAR3 was assessed in vitro. SKOV3 and OVCAR3 cells were plated on Matrigel coated membranes of invasion chambers and subsequently treated with ML246 for 72 h. ML246 significantly attenuated invasion of both cell lines as there were less cells that invaded through the membrane (Fig. 2a and b). The significant reduction in the number of cells on the underside of the membrane was not due to decreases in cell viability under these conditions (Fig. 2a and b).

### Effect of ML246 on ovarian cancer xenograft growth and spread

**Fig. 3:**
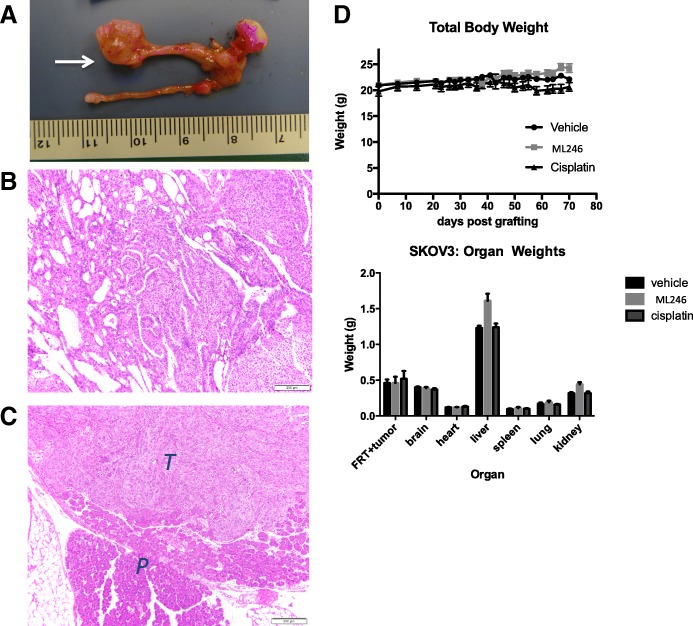
Effect of ML246 on SKOV3 cells grafted orthotopically on the ovary. SKOV3 cells in collagen pellets were implanted under the bursa of the ovary and **a** allowed to grow for 3 weeks (tumor at arrow). Mice were treated with vehicle or 25 mg/kg ML246 for 7 weeks (Monday-Friday treatment schedule). **b** Histopathology evaluation was done to validate the tumor features of the xenografts. **c** Tumor lesions outside of the ovary were collected and assessed by H&E to confirm the presence of tumor (*T =* tumor, *P* = pancreas). **d** Body weights were measured three times per week and E) organ weights were measured at the end of the treatment period to determine toxicity of the treatments

The SKOV3 cell line was xenografted under the ovarian bursa of mice and the effect of ML246 was assessed. Mice were treated with vehicle, cisplatin or ML246 and at the end of a 7-week treatment schedule, the primary tumor size was measured, then excised and analyzed by hematoxylin and eosin (H&E). Growth of SKOV3 tumors at the primary site was apparent (Fig. 3a; arrow). The tumor appeared as an endometrioid carcinoma given its cribriform and solid architecture, cytologic features including columnar cells, and mitotic index (Fig. 3b). There was high variability in tumor size at the ovary within each treatment group and thus no significant difference in tumor volume was noted between the treatments. Tumor lesions were also found in the abdominal cavity away from the primary site and confirmed by H&E staining in all treatment groups (Fig. 3c, Table 1). The number of extra-ovarian lesions differed between the treatment groups. Excluding mice that died, eight out of 11 (73%) mice had lesions outside of the primary site in the vehicle treated group (Table 1). In the cisplatin-treated group, one mouse did not have tumor on the primary site or anywhere else and overall, 3 out of 9 mice (33%) had lesions outside of the ovary. In the ML246 group, 4 out of 9 mice did not have tumor at the primary site and among these 4, 2 mice did not have tumor anywhere in the abdominal cavity. Overall, 3 out of 9 mice (33%), had lesions outside of the ovary. The difference in the incidence of tumors outside of the primary site between the groups was statistically significant (*p* = 0.012). No obvious toxicities of ML246 and cisplatin were observed in the mice as total body weights and organ weights did not differ between treatment groups with the exception of a slight increase in liver weight with ML246 treatment (Fig. 3d).

**Table 1:** Effect of ML246 and cisplatin on SKOV3 xenograft growth and spread

Mouse ID	Treatment	Primary tumor volume(mm^3^)	Lesions outside of primary site (#)	Total # extraovarian lesions
201	cisplatin	195.7		0
203	cisplatin	738.2		0
204	cisplatin	70.3		0
205	cisplatin	287.3		0
207	cisplatin	208.9	back/subQ (1)	1
211	cisplatin	25.9	back/subQ (1)	1
214	cisplatin	51.2		0
217	cisplatin	NR	*mouse died*	
229	cisplatin	NT		0
234	cisplatin	93.3	back/subQ (1)	1
206	vehicle	small	back/subQ (1)	1
208	vehicle	NR	*mouse died*	
209	vehicle	172.6	back/subQ (1)	1
210	vehicle	118.3		0
212	vehicle	11.7	pelvis (1); back/subQ (1)	2
221	vehicle	3.4		0
222	vehicle	35.9	cecum (1);	1
224	vehicle	323.8	diaphram (2); pelvis (1); bowel (1); back/subQ (1); mesentery (1)	6
225	vehicle	184.4	back/subQ (1)	1
223	vehicle	212.0		0
230	vehicle	234.0	bowel (2)	2
233	vehicle	small	pelvis (1)	1
202	ML246	NR	*mouse died*	
213	ML246	14.3		0
215	ML246	NT		0
216	ML246	140.7	back/subQ (1)	1
218	ML246	NR	*mouse died*	
219	ML246	114.6	back/subQ (1)	1
220	ML246	917.4		0
226	ML246	NT	back/subQ (2)	2
227	ML246	NR	*mouse died*	
228	ML246	NT		0
231	ML246	NT		0
232	ML246	213.3		0

*NT* No tumor, *SubQ* Subcutaneous, *NR* Not recorded

**Fig. 4:**
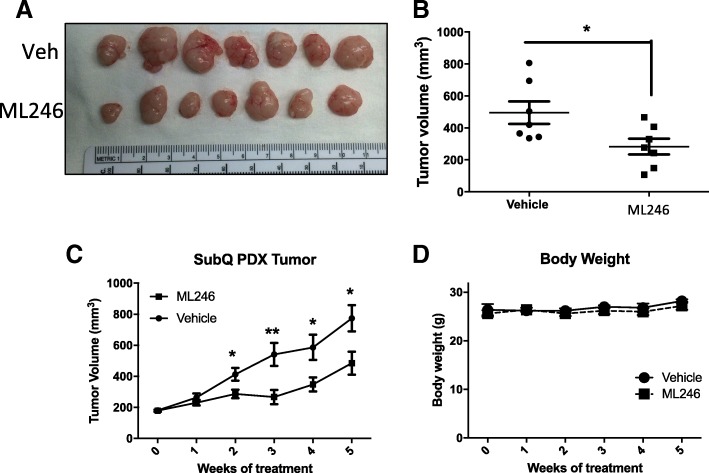
Effect of ML246 on growth of ovarian cancer PDX tumors grafted subcutaneously The OVCA4P PDX line from the third passage was implanted subcutaneously on the right and left flanks of the mice and allowed to grow for 3 weeks after which time mice were treated with Vehicle or 15 mg/kg ML246 for 5 weeks (Monday-Friday treatment schedule). Tumor growth was measured with digital calipers weekly. **a** Tumors at the end of the 5 week treatment are shown and **b** and **c** mean + SEM of tumor volumes in vehicle and ML246 treated mice are shown. **d** Body weights were measured weekly to monitor toxicity of the treatments

An ovarian cancer PDX line that was previously established in the lab [17] was grafted subcutaneously in the right and left flanks of the mouse to monitor growth of the tumors. The mean tumor volumes were significantly smaller at the end of the treatment period with ML246 compared to vehicle (Fig. 4a, b). Over a 5-week treatment period, ML246 attenuated growth of subcutaneous tumors (Fig. 4c). Body weight of the mice did not change indicating no apparent significant toxicity of ML246.

## Discussion

Platinum-based chemotherapy has been the mainstay of treatment paradigms for ovarian cancer, however, the disease remains highly lethal. Effective treatments, singular or in combination, that could significantly extend patient survival continues to be a key interest of research. Here we report that an anti-cancer small molecule, ML246, has the ability to attenuate growth and progression of ovarian cancer.

Cancer cells are highly heterogeneous among different individuals, among the affected organs of the same patient, and even within the same primary tumor. Although many cellular pathways and mutations have been shown to contribute to tumorigenesis and progression, none have been found to be singularly necessary for metastatic transformation. Rather, a complex cellular structure may better reflect tumor behavior than a single gene or gene product. The PNC, a nuclear body, forms primarily in a subset of cancer cells and has been hypothesized to be a surrogate marker for metastatic behavior of cancer cells [6–9, 14]. In breast cancer, the prevalence of PNC was significantly correlated to stage and shows a stepwise increase from a median of 23% in primary tumors to approximately 100% in distant metastases [6, 9]. Similarly, PNC prevalence was associated with disease progression in colorectal carcinoma as high PNC prevalence was associated with poor patient outcome [8, 9]. This is the first report on PNC prevalence in ovarian carcinoma and interestingly, a higher PNC prevalence was observed in the OVCAR3 cell line derived from a high grade serous carcinoma, compared to the SKOV3 cell line which originated from an endometrioid ovarian carcinoma.

ML246 was identified from a high throughput screen of compounds that reduced PNC prevalence [14, 18]. Here we tested the effects of ML246 on ovarian cancer growth, invasion and spread. Tumor growth was assessed in subcutaneous grafts of SKOV3 cells or PDX tissues. ML246 significantly attenuated growth of the PDX tumor while SKOV3 xenografts were unaffected. SKOV3 tumor sizes were highly variable and any affect of ML246 did not reach statistical significance. Grafting the human ovarian cancer cells under the bursa of the mouse ovary provided an orthotopic environment most similar to the primary disease site in humans. In addition, this site provided a niche to allow for peritoneal spread from the ovary to other intra-abdominal organs. The incidence of lesions outside of the grafting site in the ML246-treated mice carrying SKOV3 xenografts compared to the vehicle-treated mice was lower. In addition, a pilot study was done, grafting patient tumors (same PDX line used in the subcutaneous model) under the ovarian bursa and similarly, ML246 treatment decreased the total number of lesions outside of the ovary (data not shown). The decrease in the number of lesions outside of the primary site could be due to the inability of tumor cells to metastasize, or the inability of tumor cells to adhere and propagate in a new microenviroment, distant from the primary tumor source. While the presence of tumor tissues outside of the ovarian bursa is suggestive of metastasis, it is noteworthy that an incision was made to place tumor cells under the bursa. It is possible that tumor cells escaped from the surgical site making it difficult to distinguish true metastasis (invasion and migration) from displacement, and thus a technical limitation of the intrabursal model.

As ML246 is an investigational agent, there was limited pharmacokinetic data, and the ideal dose for our studies was unknown. Although the therapeutic index still remains undefined, a higher dose of ML246 may have caused a more robust response in our systems. Furthermore, pathologic sectioning was done on visible tumor deposits and grossly abnormal organs with up to three sections analyzed. Additional metastatic lesions could have gone undetected. Ultrafine sectioning of the whole organ would have given a more thorough measurement of tumor cells outside the ovary.

ML246 has not been studied at the clinical level for any tumor type although there are plans to move ML246 into human trials. Given its ability to inhibit tumor growth and metastases in preclinical models, one could foresee using this in first line, maintenance and recurrent settings, especially if it is well tolerated. Additional preclinical studies to evaluate the efficacy of ML246 in combination with platinum and/or taxane chemotherapy, would be warranted. Our data demonstrate that ML246 is as effective as cisplatin in inhibiting ovarian cancer growth and thus could be considered in platinum-resistant disease.

Our study reports for the first time, the effects of ML246, an inhibitor of PNC, in ovarian cancer. ML246 decreased invasive ability of ovarian cancer cell lines, attenuated tumor growth of human xenografts of ovarian cancer, and decreased abdominal spread of xenografts. These data warrant additional investigation into the therapeutic potential of ML246 for ovarian cancer as well as its mode of action. Given the significant heterogeneity and metastatic potential of ovarian cancer [20], the PNC structure may be a reliable target. ML246 is a promising compound that targets PNC and should be studied in greater detail in ovarian cancer.

## Conclusions

PNC’s are present in ovarian cancer cells and treatment with ML246 decreases invasion in vitro and tumor growth and spread in vivo. Additional studies will determine the efficacy of ML246 as an inhibitor of metastatic disease in ovarian cancer and to determine its precise mechanism of action.

## Abbreviations

CO_2_: Carbon dioxide
DAPI: 4′,6-diamidino-2-phenylindole
DMEM: Dulbecco’s modified Eagle’s medium
DMSO: Dimethyl sulfoxide
DNA: Deoxyribonucleic acid
FBS: Fetal bovine serum
H&E: Hematoxylin and eosin
NSG: NOD-scid IL2Rγ null
PARP: Poly ADP ribose polymerase
PDX: Patient derived xenograft
PNC: Perinucleolar compartment
PTB: Polypyrimidine tract-binding protein
RNA: Ribonucleic acid
